# Corilagin suppresses RANKL‐induced osteoclastogenesis and inhibits oestrogen deficiency‐induced bone loss via the NF‐κB and PI3K/AKT signalling pathways

**DOI:** 10.1111/jcmm.15657

**Published:** 2020-07-18

**Authors:** Jinwei Lu, Chenyi Ye, Yanyong Huang, Donghui Huang, Lan Tang, Weiduo Hou, Zhihui Kuang, Yazhou Chen, Shining Xiao, Mumingjiang Yishake, Rongxin He

**Affiliations:** ^1^ Department of Orthopedic Surgery The Second Affiliated Hospital School of Medicine Zhejiang University Hangzhou China; ^2^ Orthopedics Research Institute of Zhejiang University Hangzhou China; ^3^ Department of Orthopedic Surgery The First People’s Hospital of Xiaoshan District Hangzhou China; ^4^ Department of Orthopedic Surgery Hangzhou Third Hospital Hangzhou China; ^5^ Orthopedics Department The First Affiliated Hospital of Zhejiang Chinese Medical University Hangzhou China

**Keywords:** Corilagin, NF‐κB, osteoclast, PI3K/AKT, RANKL

## Abstract

Over‐activated osteoclastogenesis, which is initiated by inflammation, has been implicated in osteoporosis. Corilagin, a natural compound extracted from various medicinal herbaceous plants, such as *Cinnamomum cassia*, has antioxidant and anti‐inflammatory activities. We found that Corilagin suppressed osteoclast differentiation in a dose‐dependent manner, significantly decreased osteoclast‐related gene expression and impaired bone resorption by osteoclasts. Moreover, phosphorylation of members of the nuclear factor‐kappaB (NF‐κB) and PI3K/AKT signalling pathways was reduced by Corilagin. In a murine model of osteoporosis, Corilagin inhibited osteoclast functions in vivo and restored oestrogen deficiency‐induced bone loss. In conclusion, our findings suggested that Corilagin inhibited osteoclastogenesis by down‐regulating the NF‐κB and PI3K/AKT signalling pathways, thus showing its potential possibility for the treatment of osteoporosis.

## 1 INTRODUCTION

Bone metabolism is the net result of bone formation and resorption.^1^, ^2^, ^3^ Over‐activated bone resorption plays a key role in osteolytic bone diseases, especially osteoporosis, Paget disease and multiple myeloma.^4^ As one of the most severe global public‐health problems, osteoporosis affects more than 1.02 billion people worldwide and imposes an enormous burden on healthcare systems.^5^ Unfortunately, effective treatments for osteoporosis still lack. Current medications, including bisphosphonates, hormones, denosumab and teriparatide, have limitations such as a high incidence of adverse effects.^6^, ^7^, ^8^, ^9^, ^10^ Thus, the screening and study of natural agents may improve osteoporosis treatment.

Over‐activated osteoclastogenesis followed by increased levels of inflammatory factors, such as nuclear factor‐κB ligand (RANKL) and tumour necrosis factor‐α (TNF‐α), is the mechanism of osteoporosis development.^11^, ^12^, ^13^ Mature osteoclasts contain three or more nuclei and are transformed from the monocyte/macrophage lineage upon stimulation by macrophage colony‐stimulating factor (M‐CSF)and RANKL.^14^ RANKL binds to RANK and recruits TNF receptor–associated adaptor molecules, activating the expression of osteoclast‐related genes and causing excessive osteoclastogenesis and bone destruction.^15^, ^16^ Considering that only osteoclasts mediate bone resorption, inhibition of osteoclastogenesis shows promising as a therapeutic strategy for osteoporosis.^17^, ^18^

Corilagin (C_27_H_22_O_18_, β‐1‐O‐galloyl‐3,6‐(R)‐hexahydroxydiphenoyl‐d‐glucose), a natural compound of medicinal herbaceous plants such as *Caesalpinia* *coriaria*, has anti‐inflammatory,^19^, ^20^ antioxidant,^21^ hepatoprotective,^22^ anti‐brain injury,^23^ antimicrobial^24^ and anti‐gastric lesion activities^25^; it also alleviated Parkinson's disease in a rat model.^26^ Moreover, Corilagin has antitumor activity against various human malignancies, especially hepatocellular carcinoma^27^, ^28^, ^29^, ^30^ and ovarian cancer.^31^, ^32^ According to previous studies, it has recently been proven that an increase in numerous kinds of inflammatory cytokines is supposed to enhance osteoclast differentiation, inducing excessive bone resorption and pathological osteoporosis.^33^, ^34^ Since Corilagin has been shown to have an excellent anti‐inflammatory effect, and its effect on osteoclast differentiation still remains unknown, and that's why we did this research.

We investigated whether Corilagin inhibited osteoclastogenesis and protected mice from osteoporotic bone loss in vivo, and identified the underlying molecular mechanism.

## 2 MATERIALS AND METHODS

### 2.1 Materials

**FIGURE 1:**
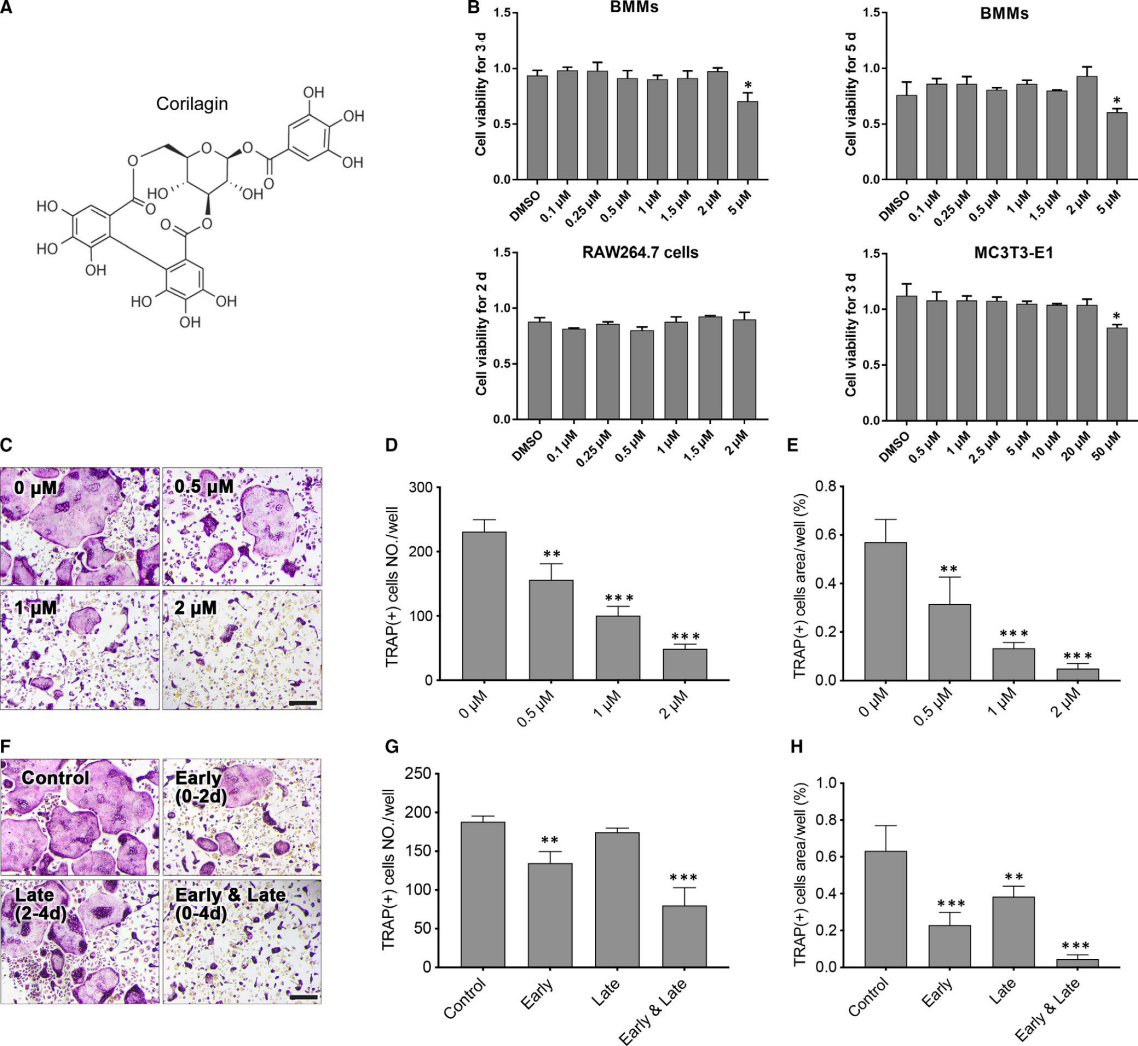
Corilagin suppressed RANKL‐induced osteoclastogenesis in vitro. A, The chemical structure of corilagin. B, The cell viability of Corilagin‐treated BMMs, RAW264.7 and MC3T3‐E1 cells was quantified by the CCK8 assay. C‐E, After treated with different concentrations of Corilagin (0, 0.5, 1, 2 μmol/L) in osteoclast medium for 4 d, TRAP staining was used to measure the number and area of mature osteoclasts. F‐H, BMMs were treated with 2 μmol/L Corilagin in the presence of 50 ng/mL M‐CSF and 100 ng/mL RANKL for 0‐2 d (early stage), 2‐4 d (late stage) or 0‐4 d (early and late stage), the numbers and area of osteoclasts were measured. Scale bar = 200 μm. All results were presented as mean ± SD. *P < .05, **P < .01, ***P < .001 vs the control group

Alpha‐modified minimal essential medium (α‐MEM) and foetal bovine serum (FBS) were purchased from Thermo Fisher Scientific (Scoresby). Corilagin (Figure 1A) with a purity greater than 98% was purchased from Tongtian Biotechnology Co. and dissolved in different concentrations of 0‐2 µmol/L with dimethyl sulphoxide (DMSO), respectively. Primary antibodies against ERK, JNK, p38, p65, IkBα, p‐ERK, p‐PI3K, TRAF6, p‐TAK1, p‐JNK, p‐p38, p‐p65, p‐IkBα, GAPDH, NFATc1/NFAT2, c‐Fos and c‐Src were purchased from Cell Signaling Technology, while second antibodies were purchased from Boster Biological Technology Co.. Recombinant RANKL and recombinant M‐CSF were obtained from Novoprotein Scientific Inc (Shanghai, China). Tartrate‐resistant acid phosphatase (TRAP) staining kit and all other reagents were purchased from Sigma‐Aldrich, unless stated otherwise.

### 2.2 Cell culture

Primary bone marrow macrophage cells (BMMs) were isolated from 6‐ to 8‐week‐old C57BL/6 mice by separating the long bones and rinsing the medullary cavity. Then, BMMs were cultured in complete α‐MEM containing 50 ng/mL M‐CSF for 5 days. To verify the toxic effect of Corilagin, BMMs were seeded into 96‐well plates at a density of 8 × 10^3^ cells/well and then treated with different concentrations of Corilagin (0, 0.1, 0.25, 0.5, 1, 1.5, 2, 5 µmol/L) for 3 and 5 days. RAW 264.7 cells were seeded into 96‐well plates with indicated concentrations of Corilagin for 2 days. Moreover, MC3T3‐E1 cells were also seeded into 96‐well plates in the presence of different concentrations of Corilagin (0, 0.5, 1, 2.5, 5, 10, 20, 50 µmol/L). After that, cell counting kit‐8 (CCK‐8, MedChemExpress) solution was added into each well in a ratio of 1:10 and cells were cultured for another 4 hours. The optical density at 450 nm was measured on the ELX808 absorbance microplate reader (BioTek).

### 2.3 TRAP staining

To investigate the inhibitory effects of Corilagin on osteoclastic differentiation, BMMs were seeded into 48‐well plates at a density of 2 × 10^4^ cells/well with α‐MEM in the presence of M‐CSF (50 ng/mL) and were incubated overnight. Afterwards, BMMs were stimulated in osteoclastogenic α‐MEM (including completely α‐MEM, 50 ng/mL M‐CSF and 100 ng/mL RANKL) with or without 2 µmol/L Corilagin for 4 days. Then, cells were stained with TRAP staining kit after fixed with 4% paraformaldehyde for 20 minutes. The number and area of true osteoclasts in each well were measured using ImageJ software (National Institutes of Health).

### 2.4 Immunofluorescence staining

BMMs were seeded at a density of 1.6 × 10^4^ cells/well in 48‐well plates and cultured with osteoclastogenic medium for 5 days until mature osteoclasts formed. Then, osteoclasts were treated with 2 µmol/L Corilagin for another 48 hours. After that, cells were fixed with 4% paraformaldehyde, permeabilized with 0.1% Triton X‐100 PBS. Then, F‐actin staining was performed with osteoclasts using rhodamine‐phalloidin for 1 hour while nuclei were stained with DAPI for 5 minutes. Fluorescence images were acquired using confocal microscopy. The number and area of F‐actin were analysed using ImageJ software.

### 2.5 Bone resorption pit assays

To determine the inhibitory effect of Corilagin on osteoclast function, BMMs were seeded on bone discs and cultured with α‐MEM (including 50 ng/mL M‐CSF) overnight. Then, cells were stimulated with osteoclastogenic medium for 6‐7 days, with or without different concentrations of Corilagin. After removing the adhered cells on bone discs, resorbed areas of three random view fields in each disc were visualized using the Hitachi (Chiyoda) S‐3700N scanning electron microscope and quantified by ImageJ software.

### 2.6 Quantitative real‐time PCR

To investigate the inhibitory effect of Corilagin on osteoclastic differentiation, BMMs were seeded in 12‐well plates at a destiny of 1 × 10^5^ cells/well and cultured with osteoclastogenic medium in the presence of different concentrations of Corilagin (0, 0.5, 1, 2 µmol/L) for 3 days. Total RNA was isolated using RNAiso reagent (Takara) and quantified using the NanoDrop 2000 (Thermo Fisher Scientific). cDNA was synthesized using previous total RNA in a 20 µL and then used for quantitative real‐time RT‐PCR. GAPDH was used for the standard of gene expression levels, and all reactions were repeated for at least three times. The murine primer sequences were as follows: GAPDH (forward: 5′‐ACCACAGTCCATGCCATCAC‐3′; reverse: 5′‐TCCACCACCCTGTTGCTGTA‐3′), TRAP (forward: 5′‐CACTCCCACCCTGAGATTTGT‐3′; reverse: 5′‐CCCCAGAGACATGATGAAGTCA‐3′); vacuolar‐type H+‐ATPase (V‐ATPase) a3 (forward: 5′‐GCCTCAGGGGAAGGCCAGATCG‐3; reverse: 5′‐GGCCACCTCTTCACTCCGGAA‐3′), NFATc1 (forward: 5′‐CCGTTGCTTCCAGAAAATAACA‐3′; reverse: 5′‐TGTGGGATGTGAACTCGGAA‐3′), cathepsin K, (forward: 5′‐CTTCCAATACGTGCAGCAGA‐3′; reverse: 5′‐TCTTCAGGGCTTTCTCGTTC‐3′), DC‐STAMP (forward: 5′‐AAAACCCTTGGGCTGTTCTT‐3′; reverse: 5′‐AATCATGGACGACTCCTTGG‐3′), MMP‐9 (forward: 5′‐CAAAGACCTGAAAACCTCCAA‐3′; reverse: 5′‐GGTACAAGTATGCCTCTGCCA‐3′).

### 2.7 Western blotting

To verify the effects of Corilagin on multiple signalling pathways, RAW264.7 macrophages were seeded into 6‐well plates at a destiny of 8 × 10^5^ cells/well. After cultured with complete α‐MEM for 24 hours, RAW264.7 were pre‐treated with or without 2 µmol/L Corilagin for another 4 hours and then stimulated with 100 ng/mL RANKL for 0, 5, 10, 20, 30 and 60 minutes, respectively. As to investigate the inhibitory effect of Corilagin on protein expression, BMMs were also seeded into 6‐well plates at a destiny of 2 × 10^5^ cells/well and stimulated with 100 ng/mL RANKL for 0, 1 and 3 days, with or without 2 µmol/L Corilagin. To detect the level of RANKL/OPG, MC3T3‐E1 cells were seeded into 12‐well plates and incubated with different concentrations of Corilagin (0, 5, 10 and 20 µmol/L) for 3 days. After washed with 10% PBS twice, total proteins were extracted using radioimmunoprecipitation (RIPA) lysis buffer containing protease and phosphatase inhibitor cocktails (Fude Biologic Technology, Hangzhou, China). Then, the extracted proteins (20 µg/lane) were separated by 10% SDS‐PAGE and transferred onto PVDF membranes (Millipore, Sigma) for 2 hours. Afterwards, membranes were blocked with 10% milk and washed by Tris‐buffered saline (including 0.1% Tween) for three times (each time for 10 minutes). Membranes were incubated with primary antibodies at 4°C overnight and washed with Tris‐buffered saline as previous. Membranes subsequently incubated with secondary antibodies for another 2 hours and washed by Tris‐buffered saline before detecting imunoreactive bands on the Bio‐Rad (Hercules) XRS chemiluminescence detection system and analysing on image software.

### 2.8 Establishment of ovariectomy‐induced osteoporosis mice model and bone histomorphology staining

Twenty 8‐week‐old female C57BL/6 mice were purchased from SLAC Laboratory Animal (Shanghai) and randomly divided into four groups: sham‐surgery group, ovariectomy (OVX) group, low‐dose (LD) group (OVX with Corilagin 4 mg/kg treatment) and high‐dose (HD) group (OXV with Corilagin 20 mg/kg treatment). OVX murine models were established according to previous studies^9^ and were approved by the Animal Care and Use Committee of Second Affiliated Hospital of Zhejiang University School of Medicine. Corilagin was firstly dissolved in DMSO at 100 mg/mL. Then, Corilagin with DMSO was dissolved in PBS at 0.4 and 2 mg/mL, respectively, and the injection volume was 10 mL/kg. One week after ovariectomy, all mice in the OVX + HD group were intraperitoneally injected 20 mg/kg Corilagin three times a week, whereas the mice in the OVX + LD group were intraperitoneally injected 4 mg/kg Corilagin. Meanwhile, equal volume of PBS containing DMSO was injected into the sham and OVX mice as a control. Two months later, all mice were euthanized by overusing anaesthesia. Bilateral femurs were separated and fixed in 4% paraformaldehyde solution for 2 days. Same number of femurs in each group were scanned by microcomputed tomography (micro‐CT; Scanco Medical, Bassersdorf, Switzerland). Bone volume/tissue volume (BV/TV), Conn.D, trabecular number (Tb.N), trabecular thickness (Tb.Th) and trabecular separation (Tb.Sp) were also quantified to evaluate the structure of the femurs. All specimens were decalcified with 10% EDTA for two months and subsequently analysed for micromorphological staining.

### 2.9 ROS quantification

Cellular reactive oxygen species (ROS) levels play an important role in osteoclast differentiation. To determine whether ROS levels changed during Corilagin treatment, RAW264.7 cells were pre‐treated with or without Corilagin for 24 hours. Then, cells were cultured with 10 mmol/L 2′,7′‐dichlorodihydro‐fluorescein diacetate (Beyotime Biotechnology) for 20 minutes in the dark. After that, cells were stimulated with 100 ng/mL RANKL for 30 minutes, and the intracellular dichlorofluorescein intensity was analysed using a multimodal microplate reader (SpectraMaxM5; Molecular Devices).

### 2.10 ALP and Alizarin red staining assay

To determine whether Corilagin had a promising effect on osteoblast formation, MC3T3‐E1 cells were seeded into 12‐well plates at a density of 6 × 10^4^ cells/well and then treated with different concentrations of Corilagin (0, 5, 10, 20 μmol/L) in osteogenic medium for 3 or 12 days, respectively. After that, the cells cultured for 3 days were performed with ALP staining while the cells cultured for 12 days were identified with Alizarin red staining. After that, all plates were photographed using a high‐resolution microscope (Leica).

### 2.11 Statistical analysis

Experimental data are presented as mean ± SD. At least three times independent experiments were repeated. Statistical criteria verified with Student's *t* test and one‐way ANOVA *P* ≤ .05 proved a statistical difference.

## 3 RESULTS

### 3.1 Selected concentrations of Corilagin had no toxic effect on BMMs, RAW 264.7 and MC3T3‐E1 cells

To investigate the toxic effect of Corilagin, BMMs were seeded into 96‐well plates at a destiny of 8 × 10^3^ cells/well and incubated with complete α‐MEM including different concentrations of Corilagin (0, 0.1, 0.25, 0.5, 1, 2 and 5 µmol/L) for 3 and 5 days. RAW 264.7 and MC3T3‐E1 cells were cultured with complete α‐MEM for 2 and 3 days, respectively. After fixed with CCK‐8 reagent at a concentration of 1:10, signal intensity was detected using Bio‐Rad's ChemiDoc XRS System and it proved that the above concentrations of Corilagin had no toxic effect on BMMs, RAW 264.7 and MC3T3‐E1 cells (Figure 1B).

### 3.2 Corilagin inhibited RANKL‐induced osteoclastogenesis

To identify the inhibitory effect of Corilagin on osteoclastogenesis, BMMs were cultured with osteoclastic medium and 2 µmol/L Corilagin for 4 days. The results showed that Corilagin had a significant inhibition on the TRAP‐positive osteoclasts differentiation, which was proved by the decreased number and area of mature osteoclasts (Figure 1C‐E). To verify which stage was significantly inhibited by Corilagin, BMMs were stimulated by osteoclastic medium supplemented with 2 µmol/L Corilagin at early stage (0‐2 days), late stage (2‐4 days) and early and late stages (0‐4 days). Corilagin noticeably reduced the area and number of mature osteoclasts at early stages, whereas Corilagin had little effect at late stage (Figure 1F‐G). The result confirmed that Corilagin inhibited osteoclast differentiation and exerted inhibition mainly at early stage.

### 3.3 Corilagin suppressed F‐actin ring formation and osteoclastic bone resorption in vitro

**FIGURE 2:**
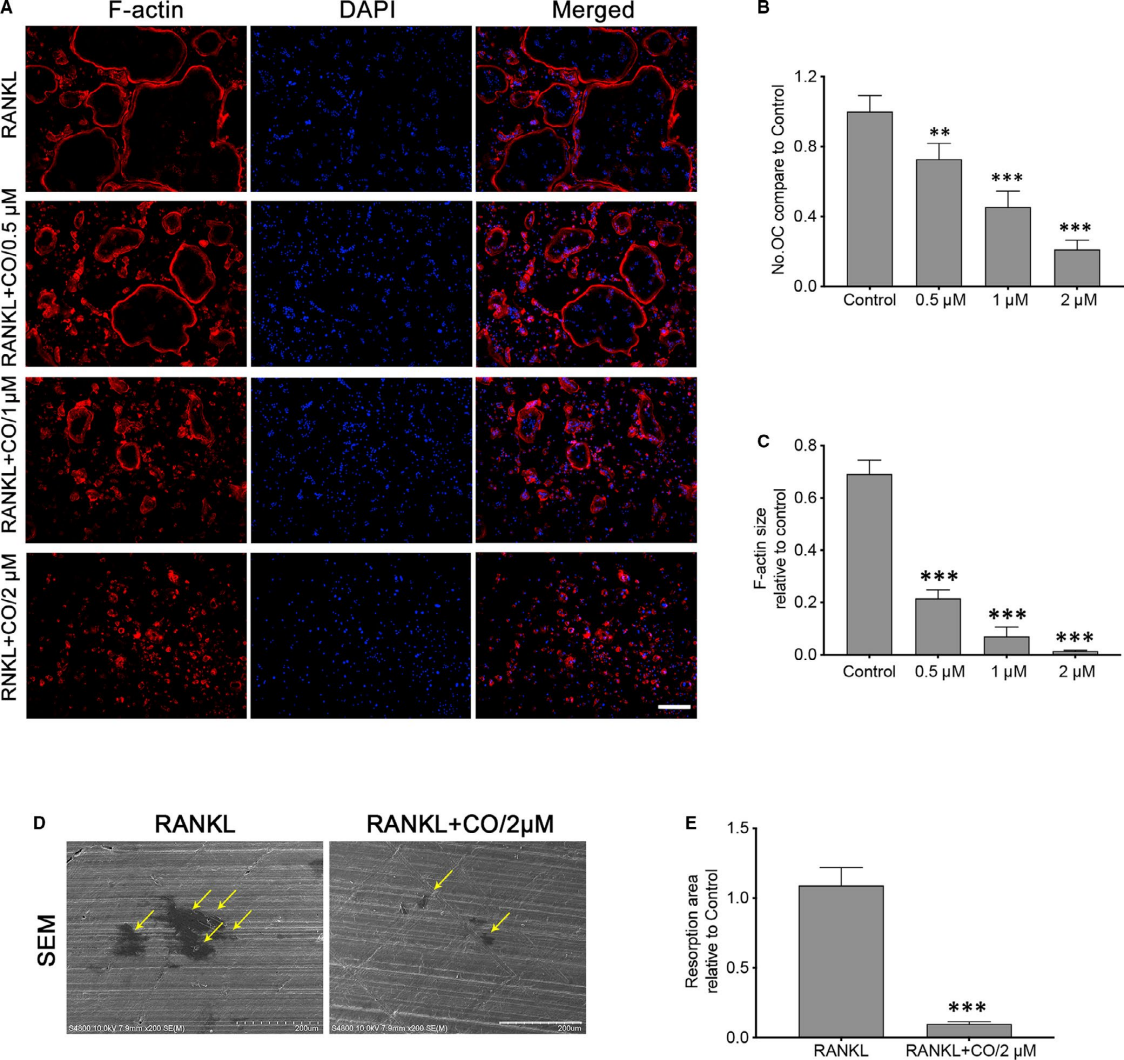
Corilagin suppressed F‐actin ring formation and osteoclastic bone resorption in vitro. A‐C, BMMs were seeded into 48‐well plates with osteoclast differentiation medium for 5 d and then cultured with different concentrations of Corilagin for another 48 h. After cells were fixed and stained, the F‐actin size and numbers of mature osteoclasts were visualized using microscopy, Scale bar = 200 μm. D‐E, BMMs were also seeded onto bone discs with or without 2 μmol/L Corilagin for 6‐7 d, and the bone resorption pits were quantified via SEM, Scale bar = 200 μm. All results were presented as mean ± SD. CO, Corilagin; OC, osteoclasts. *P < .05, **P < .01, ***P < .001 vs the control group

To investigate the inhibitory effect of Corilagin on osteoclast function, F‐actin immunofluorescence staining assays and bone resorption pit assays were presented. We first verified the effect of Corilagin on F‐actin formation. As an important indicator to evaluate the adhesion to bone and bone resorption,^17^ significant morphologic decrease in mature osteoclast size was performed after treated with 2 µmol/L Corilagin for 48 hours compared to untreated cells, which meaned F‐actin formation was attenuated (Figure 2A‐C). We next determined the effect of Corilagin on bone resorption. The results indicated that the number and area of resorption pits were markedly decreased (Figure 2D‐E). These data confirmed that Corilagin could obviously inhibit osteoclast function and the inhibitory effect may be attributed to the influence of the actin cytoskeleton.

### 3.4 Corilagin down‐regulated osteoclast‐related mRNA expression and reduced RANKL‐induced ROS production

**FIGURE 3:**
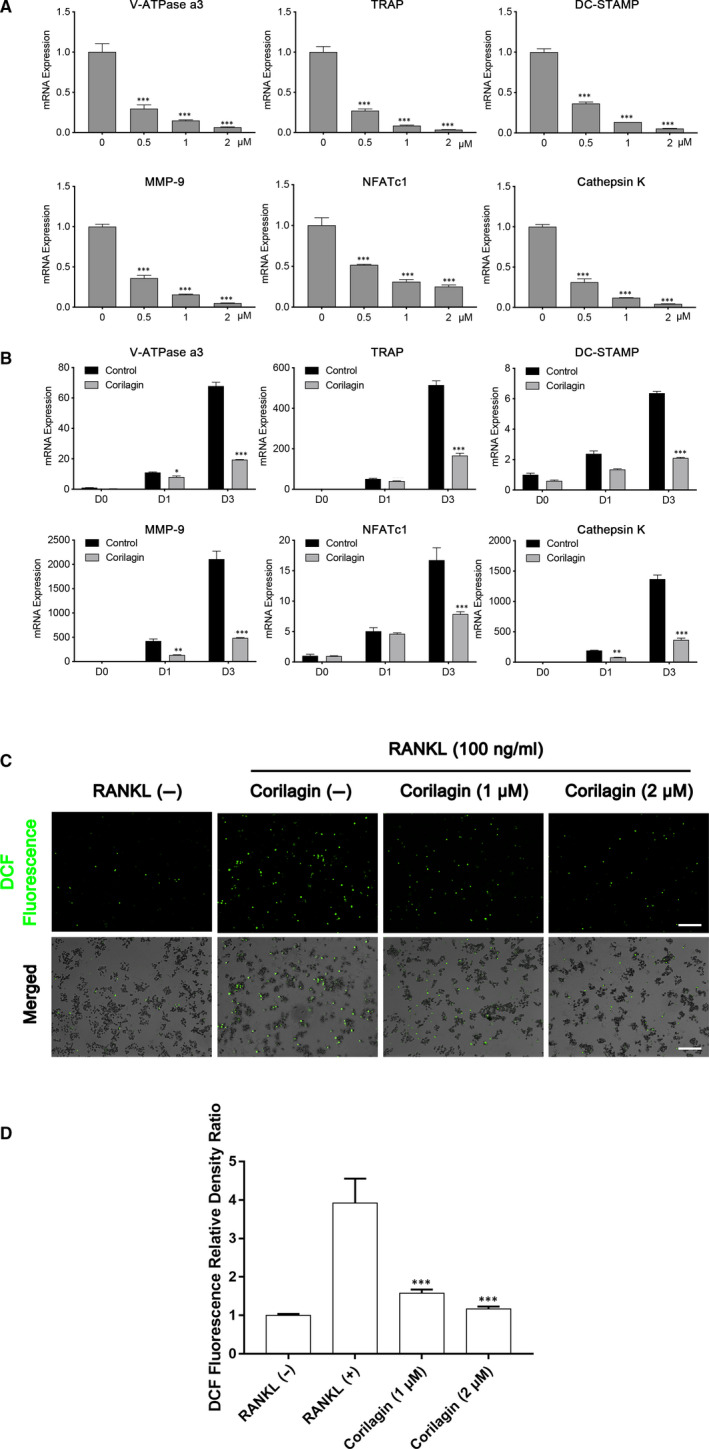
Corilagin attenuated the mRNA expression of osteoclast‐related genes, and reduced RANKL‐induced ROS production in vitro. A, BMMs were cultured with various concentrations of corilagin (0, 0.5, 1 and 2 μmol/L) in osteoclast medium for 3 d. B, Equal number of BMMs was cultured with osteoclast medium while treated with or without 2 μmol/L Corilagin for 0, 1 and 3 d. Total RNA was isolated and then quantified using qPCR. C‐D, The levels of RANKL‐induced ROS production were decreased after Corilagin treatment. Scale bar = 200 μm. All Results are performed as mean ± SD; *P < .05, **P < .01, ***P < .001 vs the control group

To determine whether Corilagin exert an inhibitory effect on mRNA expression during osteoclast differentiation, the mRNA expression levels of osteoclast‐related genes were quantified using quantitative real‐time RT‐PCR. A variety of osteoclast‐related marker genes including TRAP, DC‐STAMP, cathepsin K, MMP‐9, V‐ATPase a3 and NFATc1 were studied.^15^ Noticeably, Corilagin down**‐**regulated the expression of these genes in a dose‐dependent manner (Figure 3A) and time‐dependent manner (Figure 3B). The data indicated that Corilagin suppressed osteoclast‐related gene expression.

The levels of RANKL‐induced ROS production were evaluated using an ROS assay kit. All results demonstrated that intracellular ROS levels increased by RANKL stimulation, and significantly decreased after treated with different concentrations of Corilagin (Figure 3C,D).

### 3.5 Corilagin blocked NF‐κB and AKT signalling pathways and suppressed NFATc1 induction

To verify three major signalling pathway (MAPK, NF‐κB and AKT signalling) by which Corilagin exerted an inhibitory effect to the induction of NFATC1 and c‐Fos levels,^2^ the phosphorylation levels of JNK, ERK, p38, p65, AKT and IκBα were detected with the use of RAW264.7 cells treated with or without 2 µmol/L Corilagin. Nevertheless, the phosphorylation of JNK, ERK and p38, which also belong to MAPK family, exhibited no fundamental change. As a crucial role during osteoclast differentiation, RANKL‐induced NF‐κB signalling pathway was inhibited by Corilagin. Then, we investigated the phosphorylation expression level of IκBα, an inhibitor of NF‐κB, and the p‐IκBα was not affected in the presence of Corilagin.

TNFR‐associated factor 6 (TRAF6) is activated after RANKL binds to RANK and then facilitates the phosphorylation of TGF‐β‐activated kinase 1 (TAK1),^35^ which further activates NF‐κB signalling axis. In our study, we demonstrated that the levels of TRAF6 and p‐TAK1 were remarkably suppressed after Corilagin treatment.

**FIGURE 4:**
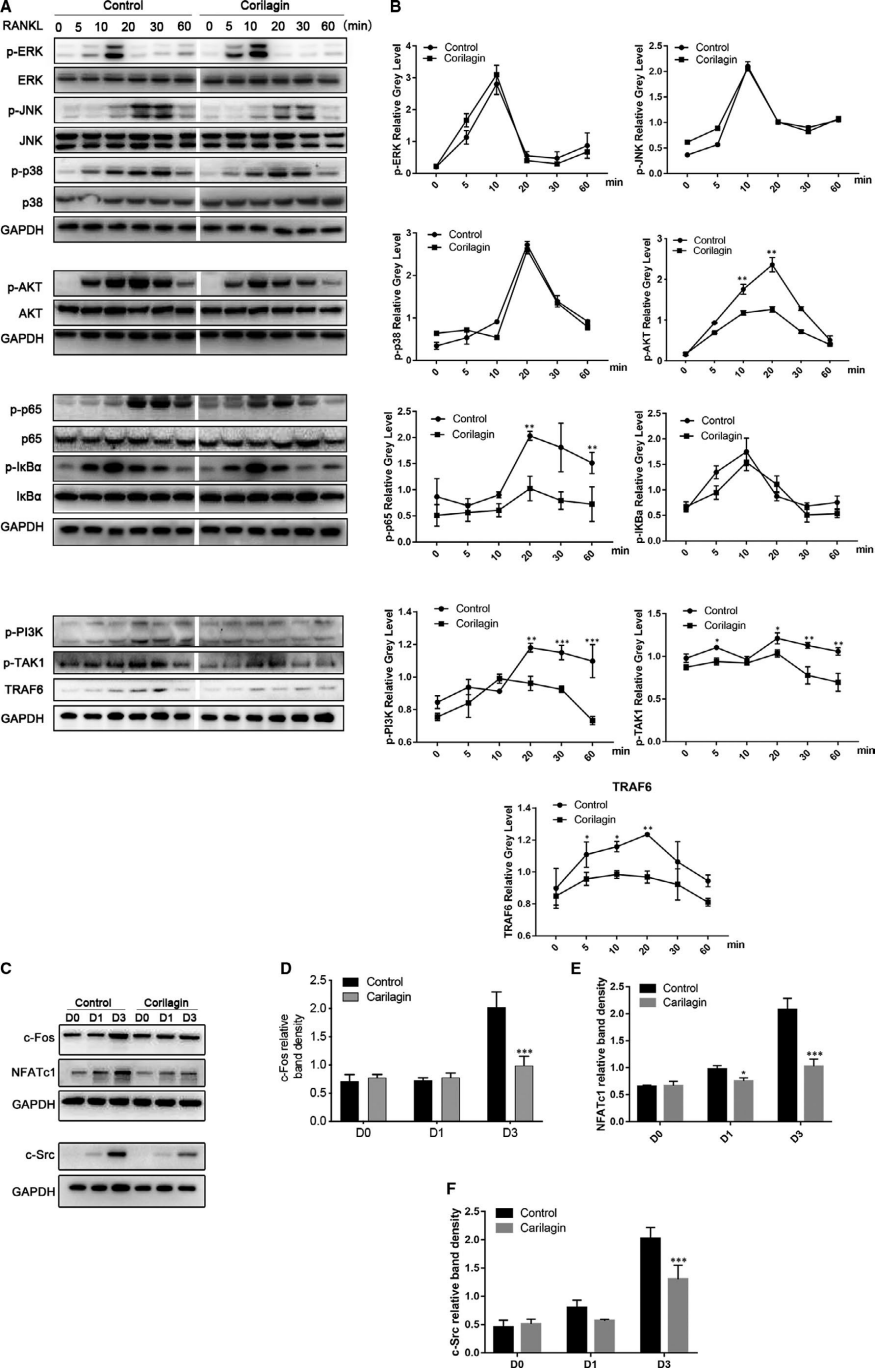
Corilagin inhibited the activation of osteoclast formation via the NF‐κB and PI3K/AKT signalling pathways. A‐B, RAW264.7 cells were seeded into 6‐well plates and cultured with α‐MEM in the presence of 50 ng/mL M‐CSF overnight. The cells were pre‐treated with 2 μmol/L Corilagin or vehicle for 6 h and then stimulated with 100 ng/mL RANKL for 0, 5, 10, 20, 30 and 60 min, respectively. Cells were lysed, and lysates were detected using Western blotting with specific antibodies. C‐F, Corilagin impaired RANKL‐induced protein expression of NFATc1, c‐Fos and c‐Src in vitro. BMMs were treated with or without 2 μmol/L Corilagin in the presence of 50 ng/mL M‐CSF and 100 ng/mL RANKL for 0, 1 and 3 d, and total cell lysates were analysed with the same way above. All results are performed as mean ± SD; *P < .05, **P < .01, ***P < .001 vs the control group

As the PI3K/AKT signalling pathway is equally important for osteoclast differentiation, we tested whether Corilagin could inhibit PI3K/AKT activation. And the phosphorylation levels of PI3K and AKT were obviously decreased during Corilagin stimulation. These data confirmed that Corilagin inhibited NF‐κB and AKT activity during osteoclast formation (Figure 4A,B).

NFATc1 and c‐Fos are regarded as the crucial regulator in the process of osteoclast differentiation.^17^ To investigate the exact molecular mechanism by which Corilagin affects osteoclast differentiation, we then determined whether Corilagin could suppress the induction of NFATc1 by detecting the protein expression of NFATc1 during osteoclast formation. The results demonstrated that NFATc1 levels changed in a time‐dependent manner and were significantly suppressed in the presence of Corilagin. As previously described, c‐Src plays an essential role in cell growth and proliferation. However, it exerts more effects in regulating osteoclast activity than enhancing cell growth. And we verified that Corilagin inhibited the levels of c‐Src during osteoclastogenesis. Furthermore, the expression of c‐Fos, which is equally important for osteoclast differentiation and function, was also attenuated in vitro (Figure 4C‐F).

### 3.6 Corilagin prevented OVX‐induced bone loss and suppressed osteoclast activity in vivo

**FIGURE 5:**
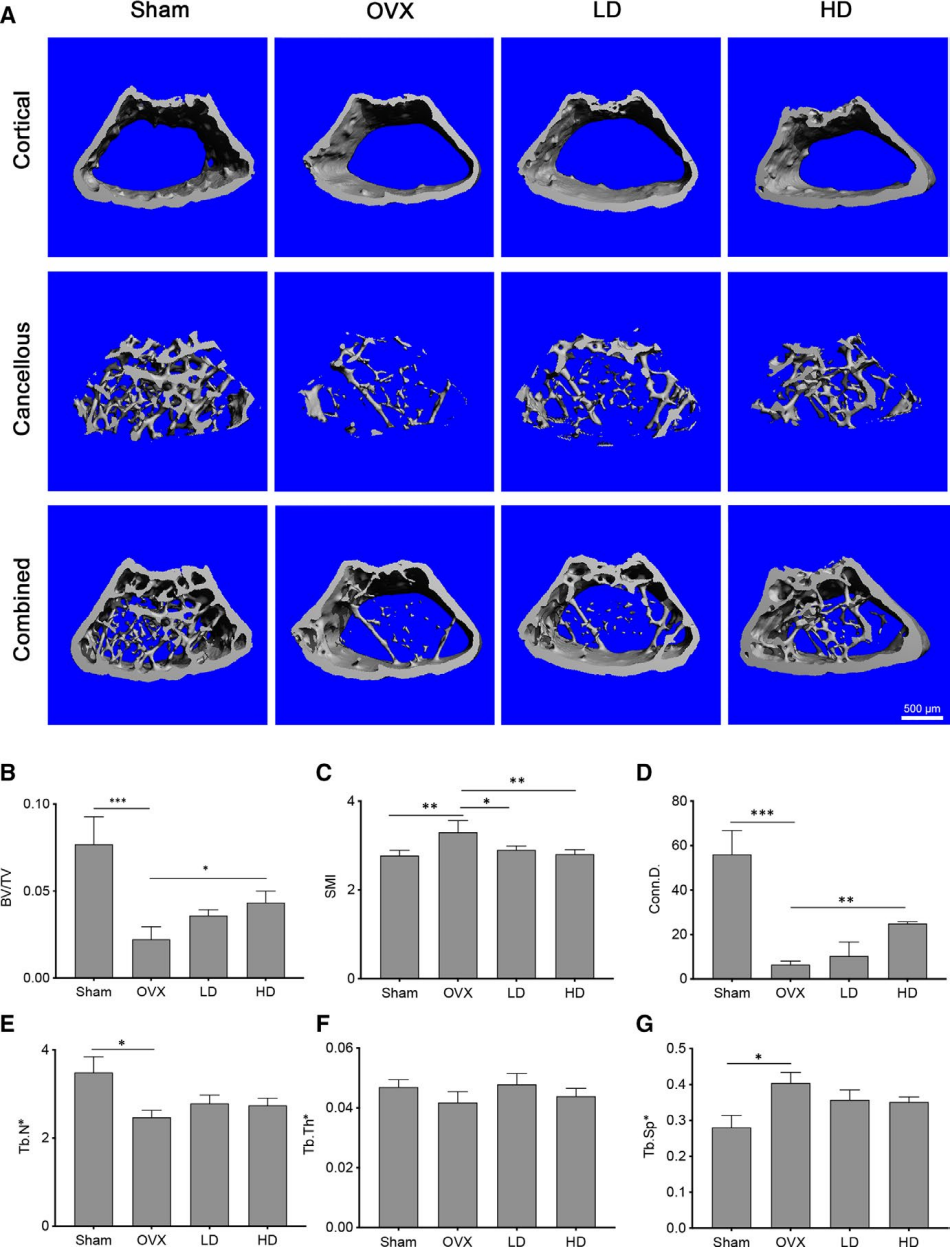
Corilagin inhibited OVX‐induced bone loss in vivo. A, Representative 3D micro‐CT images of distal femurs from four groups (Sham‐surgery groups, OVX groups, LD‐treated groups, HD‐treated groups) were performed after eight‐week surgery. B‐G, The results of BV/TV, Conn.D, SMI, Tb.N, Tb.Th and Tb.Sp from each detached femurs were analysed. Scale bar = 500 μm. All results are performed as mean ± SD. LD: low dose; HD: high dose. *P < .05, **P < .01, ***P < .001 vs the control group

We finally investigated the effects of Corilagin on bone remodelling by using a C57BL/6 mice OVX‐induced osteoporosis model. After the OVX models were established, a significant decrease in bone volume occurred mice in the OVX groups compared to those in the Sham‐surgery groups by the performance of decreased BV/TV, Tb.N, Tb.Th and Conn.D. Then, we treated mice with vehicle or different concentrations of Corilagin, respectively. According to micro‐CT results, Corilagin treatment showed significant protective effects against OVX‐induced bone volume loss especially in the high‐dose groups (20 mg/kg), while weaker effects were observed in the low‐dose groups (4 mg/kg) (Figure 5A). Increased values in Conn.D, Tb.N, Tb.Th, BV/TV and decreased numbers in SMI and Tb.Sp further confirmed our findings (Figure 5B‐G).

**FIGURE 6:**
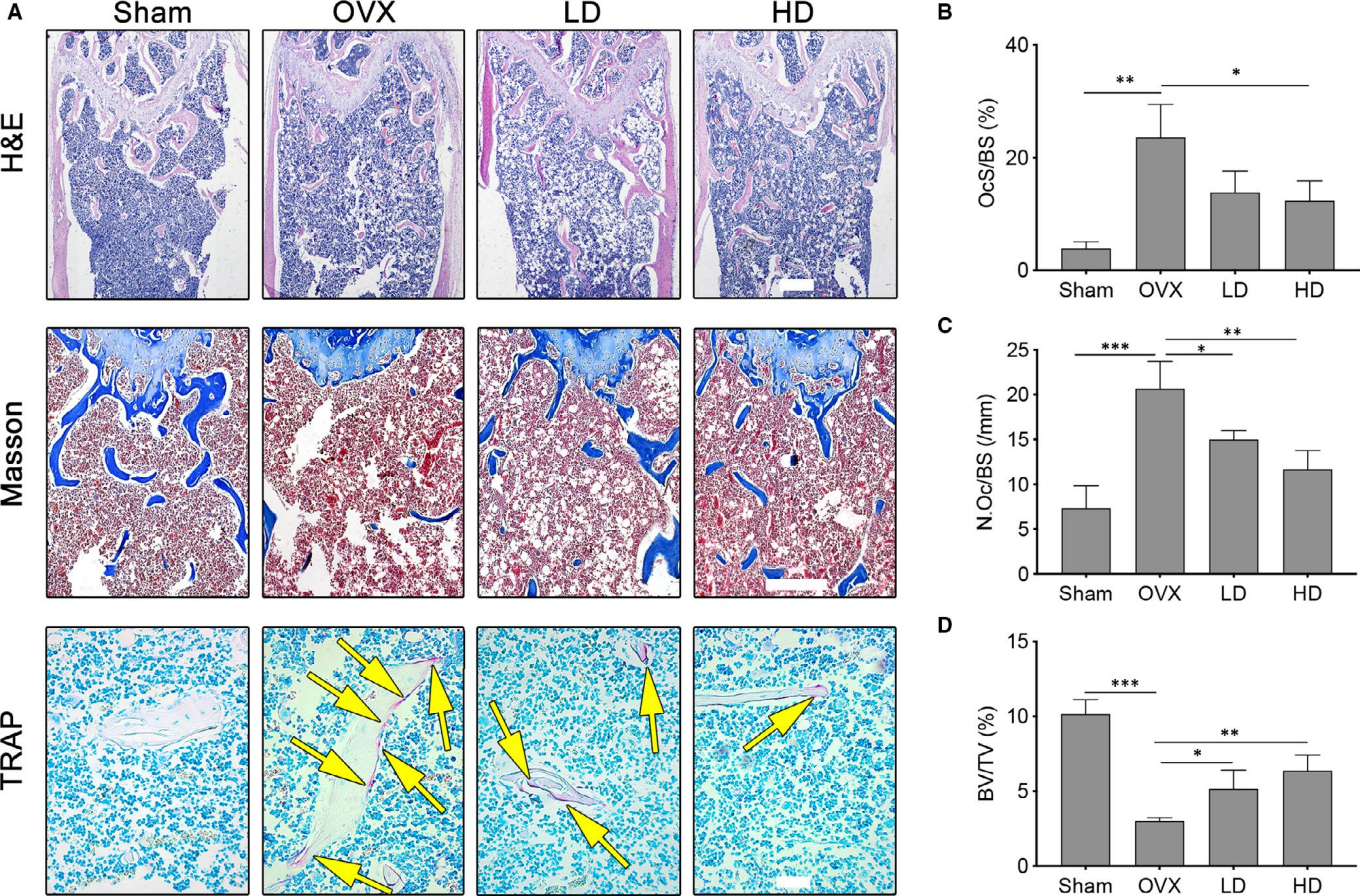
Corilagin mitigated OVX‐induced bone loss and osteoclast formation. A, H&E staining, Masson staining and TRAP staining were performed for evaluating trabecular bone of distal femurs from each group. B‐D, The Oc.S/BS, BV/TV and N.Oc/BS were quantified and analysed in each group. Scale bar (HE staining) = 500 μm; scale bar (Masson staining) = 200 μm; scale bar (TRAP staining) = 50 μm. All results are performed as mean ± SD. OcS/BS: the surface area of osteoclasts normalized to bone surface area; BV/TV: bone volume/tissue volume; N.Oc/BS: the number of osteoclasts normalized to the bone surface. *P < .05, **P < .01, ***P < .001 vs the control groups

Meanwhile, to investigate the protective effect of Corilagin on OVX‐induced osteoporosis in histological level, we evaluated distal femur via using haematoxylin and eosin staining (H&E staining), Masson staining and TARP staining. The results demonstrated that low dose and high dose of Corilagin inhibited bone mass loss. The number and size of trabecular bone memorably increased in the Corilagin‐treated groups in a dose‐dependent manner compared to the OVX groups. TRAP staining results revealed that the number and area of TRAP‐positive cells increased after ovariectomy compared with the control groups, while the number of TRAP‐positive cells displayed an obvious reduction using Corilagin treatment, which was coordinated with the above results (Figure 6A). The micromorphological quantification showed that there was a significant reduction in the surface area of osteoclasts normalized to bone surface area (OcS/BS (%)), the number of osteoclasts normalized to the bone surface (N.Oc/BS) values and increase in bone volume/tissue volume (BV/TV) value after Corilagin treatment (Figure 6B‐D). Statistical results of OcS/BS (%) and N.Oc/BS were obtained from TRAP staining images, while the data of BV/TV were obtained from H&E staining images. In summary, the results confirmed that Corilagin exactly exerted a protective effect on oestrogen deficiency‐induced bone loss in vivo.

### 3.7 Corilagin exerted no significant effects on osteogenesis and had no obvious impacts in regulating RANKL/OPG expression

**FIGURE 7:**
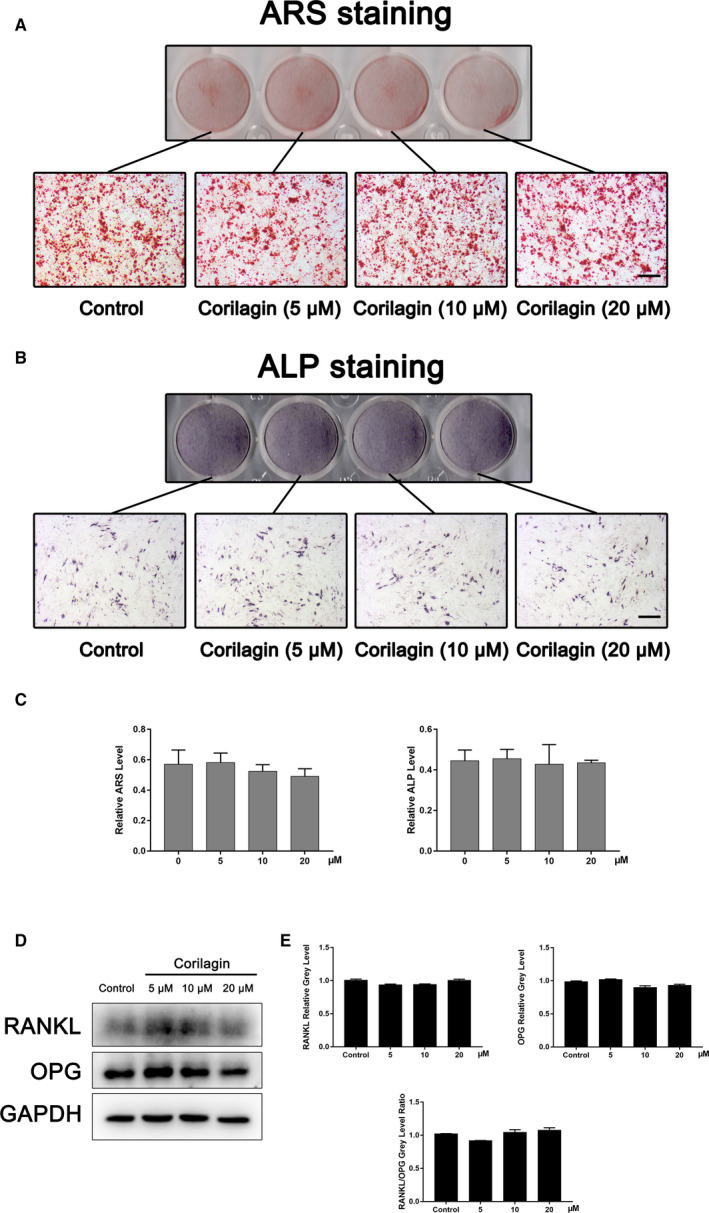
Corilagin exerted no apparent effects on osteogenesis and had no obvious impacts in regulating osteoclast activity. A‐C, MC3T3‐E1 cells were seeded into 12‐well plates and incubated with indicated concentrations of Corilagin for 3 d or 12 d, respectively. All plates were performed with Alizarin red and ALP staining. Scale bar = 500 μm. D‐E, MC3T3‐E1 cells were also treated with Corilagin for 3 d, and no significant differences occurred in the level of RANKL/OPG. *P < .05, **P < .01, ***P < .001 vs the control groups

To investigate the effect of Corilagin on osteoblast differentiation, MC3T3‐E1 cells were incubated with indicated concentrations of Corilagin in osteogenic medium, and then performed with Alizarin red and ALP staining. The results confirmed that Corilagin exerted no significant effects on osteogenesis (Figure 7A‐C).

To detect the protein level of RANKL/OPG, which leads to changes in osteoclast differentiation and function, MC3T3‐E1 cells were also seeded into 12‐well plates and cultured with different concentrations of Corilagin for 3 d. During Corilagin treatment, there have been no obvious changes in the expression of RANKL and OPG, neither did the level of RANKL/OPG (Figure 7D‐E).

## 4 DISCUSSION

As a major disease related to osteoclast overactivation, osteoporosis is a global threat to health and imposes a considerable burden on healthcare systems.^1^, ^10^ As hyperactive osteoclastogenesis and bone resorption are implicated in other pathologic orthopaedic disorders, such as rheumatoid arthritis, aseptic loosening and Paget disease,^10^, ^36^, ^37^ investigation of the regulation of osteoclastogenesis and the re‐balance of bone metabolism is important.

﻿In addition to current medications such as bisphosphonates, hormones and antibodies, certain natural ﻿compounds exhibit anti‐resorption properties. Indeed, several natural ﻿compounds, including sophocarpine and tomatidine, inhibited osteolysis and osteoporosis in animal models.^6^ Corilagin shows promise as a medicinal herbal agent against various diseases.^8^, ^30^, ^38^ Jia et al reported that Corilagin inhibited the growth of SKOv3ip ovarian cancer cells in vitro and of xenograft tumours in vivo.^30^ Reddy et al reported that Corilagin blocked hepatitis C viral replication and protected against liver damage by modulating oxidative stress.^38^ Corilagin also reportedly ameliorated nutritional steatohepatitis in vitro and in vivo.^8^ In this study, we demonstrated, for the first time, that Corilagin suppressed osteoclastogenesis in vitro and significantly inhibited oestrogen deficiency‐induced osteoporosis in a murine model. Furthermore, these effects of Corilagin were mediated by suppression of the NF‐κB and PI3K/AKT signalling pathways.

Although the therapeutic potential of diverse natural herbal compounds for osteoporosis has been investigated, most have not been well investigated, and their side effects and long‐term safety are unknown. In contrast, no toxicity of Corilagin toward normal cells and tissues has been reported.^39^ Moreover, Corilagin protects various organs, such as the liver, kidney and lung, from acute and chronic injury.^39^, ^40^, ^41^ Indeed, our data showed that Corilagin exerted little cytotoxicity against osteoclast precursors, confirming its safety and therapeutic potential.

RANKL/RANK signalling axis enhances the differentiation and function of mature osteoclasts from precursors, while osteoprotegerin (OPG) inhibits over‐activated bone resorption by preventing RANKL from binding to RANK.^42^ According to previous studies, RANKL and RANK exist in the early stage of osteogenic formation of bone marrow mesenchymal stem cells (BMSCs) and is thought to inhibit osteoblast differentiation.^43^ OPG is secreted from osteoblasts, and its down‐regulation is shown to be consistent with the up‐regulation of RANKL.^44^ In consequence, the protein level of RANKL/OPG is regarded as a critical factor of bone volume and skeletal integrity. Our study demonstrated that Corilagin had no apparent effects on the expression of RANKL and OPG.

RANKL‐induced signalling cascades play a vital role in regulating osteoclast activity.^45^ In addition, increased DC‐STAMP expression is crucial for facilitating the cell‐to‐cell fusion of precursors in response to RANKL stimulation.^14^ We found that Corilagin inhibited DC‐STAMP expression at the mRNA level and suppressed osteoclast differentiation, mainly during the early stage. The recruitment of TNF receptor–associated factors (TRAFs) is regarded as a crucial role in provoking various signalling pathways, and TRAF6 is one of the mostly important factors.^46^ After binding of RANKL to RANK, TRAF6 is activated and forms a complex with TAK1.^47^ Activated TAK1 subsequently phosphorylates the IKK/IκBα and MAPK pathways, leading to activation of the NF‐κB pathway and activated protein 1 (AP‐1).^17^, ^18^, ^48^ Our results showed that Corilagin suppressed the phosphorylation of NF‐κB but did not affect that of p38, ERK or JNK. Upon stimulation of RANKL, the PI3K/AKT signalling pathway promotes TRAF6/Src/PI3K interaction, which influences osteoclastogenesis.^4^ Our data revealed that Corilagin inhibits not only the NF‐κB but also the PI3K/AKT pathway. Moreover, some studies have identified that a novel factor named ribosomal protein S5 (RPS5) could regulate NF‐κB, MAPK and AKT signalling pathways,^49^, ^50^ which seems to be a promising target for osteoporosis treatment.

Activated NF‐κB and AP‐1 stimulate the multiplication of NFATc1 and modulate the expression of osteoclast‐related genes including TRAP, V‐ATPase a3, DC‐STAMP, MMP‐9 and cathepsin K, which influences differentiation of osteoclast precursors into mature osteoclasts.^51^ We found that Corilagin decreased NFATc1 mRNA and protein levels and the mRNA levels of the above marker genes, suggesting that Corilagin suppresses not only NFATc1 expression but also that of its downstream genes.

Several limitations of this study are notable. First, osteoporosis involves both bone destruction and bone formation. However, we explored only the protective effects of Corilagin on osteoclastogenesis and OVX‐induced bone loss; its effect on bone formation still needs evaluation. Second, although previous studies have confirmed the protective effect of Corilagin on the lung, kidney and liver, its effect on other organs needs to be investigated. Finally, the mechanism by which Corilagin suppressed the NF‐κB signalling pathway remains to be explored.

In conclusion, Corilagin inhibited RANKL‐induced osteoclastogenesis and prevented osteoporotic bone loss in vivo. These effects of Corilagin were mediated by impairment of the NF‐κB and PI3K/AKT pathways. As a result, we believe that Corilagin shows promise for the treatment of osteoporosis.

## CONFLICT OF INTEREST

The authors declare no conflict of interest.

## AUTHOR CONTRIBUTION

**Jinwei Lu:** Conceptualization (equal); Data curation (equal); Formal analysis (equal); Investigation (equal); Methodology (equal); Resources (equal); Software (equal); Supervision (equal); Validation (equal); Visualization (equal); Writing‐original draft (equal); Writing‐review & editing (equal). **Chenyi Ye:** Conceptualization (equal); Data curation (equal); Formal analysis (equal); Investigation (equal); Supervision (equal); Validation (equal); Writing‐original draft (equal); Writing‐review & editing (equal). **Yanyong Huang:** Data curation (equal); Formal analysis (equal); Investigation (equal); Resources (equal); Software (equal); Writing‐original draft (equal). **Donghui Huang:** Funding acquisition (supporting); Resources (equal); Software (equal); Writing‐original draft (equal); Writing‐review & editing (equal). **Lan Tang:** Resources (equal); Software (equal); Writing‐original draft (equal); Writing‐review & editing (equal). **Weiduo Hou:** Resources (equal); Software (equal); Supervision (equal); Writing‐original draft (equal). **Zhihui Kuang:** Resources (equal); Software (equal); Writing‐original draft (equal). **Yazhou Chen:** Resources (equal); Software (equal); Writing‐original draft (equal). **Shining Xiao:** Resources (equal); Software (equal); Writing‐review & editing (equal). **Mumingjiang Yishake:** Funding acquisition (supporting); Resources (supporting); Software (supporting). **Rongxin He:** Conceptualization (lead); Data curation (lead); Funding acquisition (lead); Investigation (lead); Project administration (lead).

## Data Availability

All the data that support the findings of this study are included in the manuscript.
